# Neovascularization Detection and Localization in Fundus Images Using Deep Learning

**DOI:** 10.3390/s21165327

**Published:** 2021-08-06

**Authors:** Michael Chi Seng Tang, Soo Siang Teoh, Haidi Ibrahim, Zunaina Embong

**Affiliations:** 1 School of Electrical and Electronic Engineering, Engineering Campus, Universiti Sains Malaysia, Nibong Tebal 14300, Malaysia; michaeltang951225@student.usm.my (M.C.S.T.); haidi@usm.my (H.I.); 2 Department of Ophthalmology, School of Medical Sciences, Health Campus, Universiti Sains Malaysia, Kubang Kerian 16150, Malaysia; zunaina@usm.my

**Keywords:** diabetic retinopathy, neovascularization detection, convolutional neural network, deep learning, computer-aided diagnosis

## Abstract

Proliferative Diabetic Retinopathy (PDR) is a severe retinal disease that threatens diabetic patients. It is characterized by neovascularization in the retina and the optic disk. PDR clinical features contain highly intense retinal neovascularization and fibrous spreads, leading to visual distortion if not controlled. Different image processing techniques have been proposed to detect and diagnose neovascularization from fundus images. Recently, deep learning methods are getting popular in neovascularization detection due to artificial intelligence advancement in biomedical image processing. This paper presents a semantic segmentation convolutional neural network architecture for neovascularization detection. First, image pre-processing steps were applied to enhance the fundus images. Then, the images were divided into small patches, forming a training set, a validation set, and a testing set. A semantic segmentation convolutional neural network was designed and trained to detect the neovascularization regions on the images. Finally, the network was tested using the testing set for performance evaluation. The proposed model is entirely automated in detecting and localizing neovascularization lesions, which is not possible with previously published methods. Evaluation results showed that the model could achieve accuracy, sensitivity, specificity, precision, Jaccard similarity, and Dice similarity of 0.9948, 0.8772, 0.9976, 0.8696, 0.7643, and 0.8466, respectively. We demonstrated that this model could outperform other convolutional neural network models in neovascularization detection.

## 1. Introduction

Diabetes causes several long-term systemic complications that have far-reaching consequences for the patients [1]. Individuals are typically diagnosed with diabetes during their most prosperous years [2]. Diabetes is becoming an epidemic on a global scale. This growth is typically faster in developed countries [3]. The etiology of this increase has been linked to behavioral changes, increased sugar consumption, sedentary lifestyle, and decreased physical activity [4,5]. According to the World Health Organization, diabetes mellitus affected approximately 422 million people in 2014. Around 5% of diabetic patients develop a significant visual acuity deficit of 5/200 or worse [6]. This condition is known as Diabetic Retinopathy (DR). It has become the leading cause of blindness in adults [7]. 

DR is caused by damage in blood vessels of the retina. It can be classified into two subtypes: Non-proliferative Diabetic Retinopathy (NPDR) and Proliferative Diabetic Retinopathy (PDR) [8]. NPDR is distinguished by microvascular leakage of the retinal blood vessels, which results in microaneurysms, exudates, and hemorrhages [9]. PDR is a progression of NPDR that involves neovascularization [10]. NPDR and PDR both carry the risk of significant vision loss [9]. However, PDR is more severe because it has the potential to develop microvascular occlusion of retinal vessels. In response, the retina develops new, delicate blood vessels. This process is called neovascularization. Vitreous bleeding can occur if these fragile new blood vessels rupture [11]. This vitreous bleeding is a dangerous condition, as the blood in the vitreous will organize and form fibrous tissue. Contraction of fibrous tissue will cause traction to the retinal layer and damage the retinal cells [12]. As a consequence, severe visual impairment may occur.

The retina is a unique site for fundus imaging and microvascular disease diagnosis [13]. Recent advances in retinal imaging have made the development of computer-aided methods for automatic retinal disease detection possible [14]. This approach has recently attracted numerous researchers to develop retinal screening systems using imaging techniques due to its low cost and scalability [15,16]. Nevertheless, it is still difficult to detect neovascularization in PDR due to its tiny size and random growth pattern. Thus, it is not easy to construct an automatic diagnosis system for PDR detection because automated disease diagnosis is ineffective in the presence of complicated health conditions [17]. Recent techniques of PDR detection are commonly based on analyzing the retinal fundus images. It typically begins with image enhancement and optic disk removal, followed by the extraction of the disease’s clinical features using image processing or machine learning techniques. 

Manual diagnostics take an excessive amount of time to complete [18]. Automated diagnosis can significantly reduce the amount of time, money, and commitment required [19]. Therefore, automated screening technologies have gained popularity in DR detection over the last few years [20]. Image recognition, interpretation, machine learning approaches, and deep learning algorithms have become popular techniques in automatic screening systems [21]. The screening systems aim to segment anatomical structures such as fovea, microaneurysms, swelling, exudates, veins, and neovascularization lesions [22]. Moreover, separating the optic disk from the abnormal lesions has also become a critical task in the screening systems [23]. 

In medical practice, several diagnostic and disease-recognition methods were used. These include fundus fluorescein angiography, direct ophthalmoscopes, indirect ophthalmoscopes, stereoscopic fundus photography, and monochromatic optical color photography [24,25]. Using these techniques, ophthalmologists can identify PDR with a sensitivity of approximately 50% [26]. However, as the number of PDR patients increases, more effort is needed to diagnose the disease. Computer-aided diagnostic systems have, therefore, been implemented to alleviate the burden on physicians. Nevertheless, these systems are not convincingly accurate to prevent defective detection [27,28]. Therefore, this study aims to improve neovascularization detection using a deep learning technique.

In recent years, deep learning has gained popularity in many application areas. For example, it has been used in the application of the Internet of Things (IoT) for malware detection [29] and super-resolution image reconstruction [30]. Deep learning is also widely used for medical purposes include COVID-19 screening [31,32], breast cancer detection [33], and bacterial shape classification [34]. In this study, deep learning is used because it can learn the features of neovascularization automatically. In contrast to conventional machine learning algorithms, manual feature extraction is required prior to training a classifier. The disadvantage is that, as the object becomes more complicated, the extraction of the features becomes more difficult. Thus, by utilizing deep learning, the complex features of the object can be automatically deduced, allowing for more accurate detection.

The main contribution to this paper is the proposal of a novel semantic segmentation convolutional neural network architecture for neovascularization detection from fundus images. The proposed network can automatically identify and segment the neovascularization pixels in the image, which is not achievable in the previously described neovascularization detection methods. 

This paper is divided into five sections. Section 2 presents the related works for neovascularization detection. Section 3 describes the proposed method and the performance evaluation. The evaluation results and discussion are presented in Section 4. Finally, conclusions are given in Section 5.

## 2. Related Works

PDR detection aims to detect abnormal blood vessels in retinal images caused by neovascularization. Numerous approaches for detecting neovascularization have been proposed in the literature. The methods can be divided into two categories: traditional and deep learning.

### 2.1. Traditional Methods

Hassan et al. [35] used conventional image processing techniques to detect neovascularization. The input fundus images are pre-processed using green channel extraction and contrast enhancement to highlight the blood vessel structures in the fundus images. Then, neutral-density filtering and morphological closing are used to extract the blood vessels. The image is then binarized using thresholding. The extracted vessels are further refined using morphological spurs, skeletonization, and thinning. Finally, neovascularization is detected by sliding a 100 × 100 pixels window through the image with extracted vessels. If a window region contains more than four blood vessels with vessel density greater than 7%, then the region is classified to contain neovascularization.

Several image features were used by Saranya et al. [36] and Ramasubramanian et al. [37] for neovascularization detection. These features include shape, brightness, position, and contrast. After they extracted the features from the fundus images, they used different classifiers for neovascularization detection. Saranya et al. [36] used a K-Nearest Neighbor (KNN) classifier, whereas a Support Vector Machine (SVM) is used by Ramasubramanian et al. [37]. Agurto et al. [38] created several multiscale representations of magnitude, frequency, and phase using multiscale Amplitude Modulation–Frequency Modulation (AM-FM) decompositions for neovascularization detection. The image representations are subsequently divided into regions of interest. Statistical features are calculated from each region of interest, and K-means clustering is then used to detect neovascularization. In another paper by Agurto [39], the AM-FM features are used together with a partial least squares (PLS) classifier for neovascularization detection. The characteristics of several neovascularization features were evaluated by Vatanparast and Harati [40]. These features include Gray-Level Co-Occurrence Matrix (GLCM), Gabor filters, AM-FM, Local Binary Patterns (LBP), and invariant LBP rotation. They showed that, among the features, the AM-FM approach is the most reliable.

Goatman et al. [41] proposed a method to detect neovascularization on the optic disk (NVD). First, they extracted the blood vessel segments using watershed lines and ridge strength measurement. Fifteen features, including shape, position, orientation, brightness, contrast, and line density, are then calculated from each segment, and an SVM is used to categorize them as normal or abnormal. Frame et al. [42] used GLCM for neovascularization textures’ analysis. Six statistics values from the GLCM are used in their proposed method. Jelinek et al. [43] performed a study of 27 fluorescein angiogram images to analyze vascular pattern characteristics to detect PDR. They segmented the image using Gabor wavelet transform and extract the area, perimeter, and five morphological features based on the derivatives-of-Gaussian wavelet-derived data to determine the presence of PDR. Nayak et al. [44] proposed a simple artificial neural network for detecting PDR using area and perimeter features extracted from the blood vessels. A dataset with 36 images was used, and they reported an accuracy of 90.91 percent. 

### 2.2. Deep Learning Methods

Neovascularization is hard to detect because it has a spontaneous growth pattern. In addition, the blood vessels that make up the lesion could be as small as one pixel wide. Therefore, several researchers have proposed to use deep learning for neovascularization detection. Deep learning, such as the convolutional neural network, has gained popularity recently and has been shown to achieve good performance in object recognition from images.

Roy and Biswas [45] suggested several novel convolutional neural networks for retinal vessel segmentation and optic disk detection. The segmented vessels are then examined to detect neovascularization using artery–vein classification. The optic disk detection is performed to identify neovascularization in the disk (NVD). Although their system is effective at detecting neovascularization, it is not entirely automated. Additional effort is needed to localize neovascularization.

Setiawan et al. [46] have implemented several pre-trained convolutional neural networks in the detection of neovascularization. These networks consisted of AlexNet, VGG16, VGG19, ResNet50, and GoogLeNet. They extracted the features from the networks and used them to train an SVM classifier to classify whether an image patch contains neovascularization. However, their approach can only determine the presence of neovascularization in an image. It is unable to pinpoint the exact location of the neovascularization lesion. 

In this paper, a novel semantic segmentation convolutional neural network architecture for neovascularization detection is proposed. The network can automatically detect and localize neovascularization lesions, which is not possible in the previously published works. We demonstrated that the proposed network could outperform other convolutional neural networks in neovascularization detection.

## 3. Methodology

**Figure 1:**
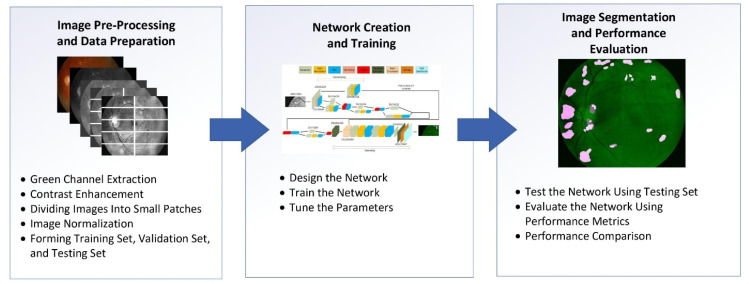
Flow chart of the methodology.

Figure 1 shows the flow of the methodology in this study. It consists of three stages: image pre-processing and data preparation, network creation and training, and image segmentation and performance evaluation. 

The image pre-processing and data preparation stage enhance the raw fundus images and crop the images into patches that are suitable to be processed by the network. In the second stage, a new semantic segmentation neural network based on the convolutional neural network is developed for neovascularization detection. The network is then trained using the prepared images, and its parameters are fine-tuned to achieve the best possible result. In the third stage, the developed network is used for neovascularization segmentation, and its performance is evaluated.

**Figure 2:**
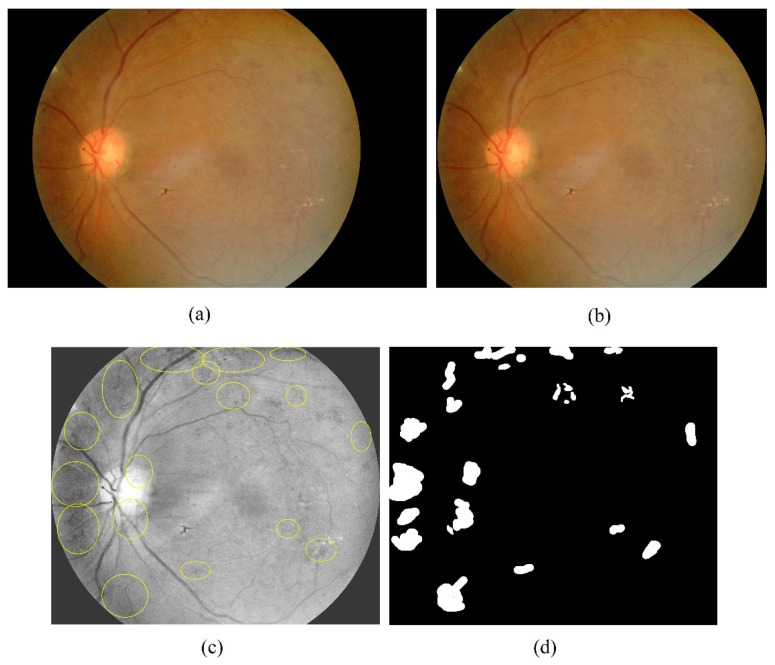
The process of ground truth generation. (**a**) original fundus image; (**b**) cropped image (**c**) labeled image by an ophthalmologist after green channel extraction and contrast enhancement; (**d**) ground truth image.

The fundus images used in this study are obtained from the Department of Ophthalmology, Health Campus, Universiti Sains Malaysia. There is a total of 20 color images, each with a resolution of 2000 × 3008 pixels. The raw images are first cropped to remove some background pixels that do not contain the retina. The cropped images have a resolution of 2000 × 2368. After green channel extraction and contrast enhancement, an ophthalmologist identified and labeled the neovascularization regions on the images. Based on the labels, a set of ground truth images are created by labeling each pixel as either neovascularization or non-neovascularization. An open-source software called Sefexa [47] is used in the labeling process and the ground truth generation. Figure 2 shows a fundus image with neovascularization and the process of creating a ground truth.

### 3.1. Image Pre-Processing and Data Preparation

**Figure 3:**
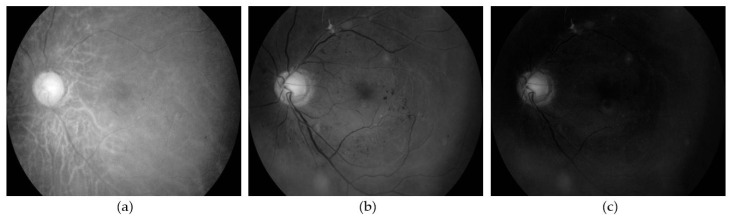
The appearance of fundus image in three separate color channels. (**a**) the red channel; (**b**) the green channel; and (**c**) the blue channel. The blood vessels appear more evident in the green channel.

Image pre-processing is required to make the neovascularization features visible in a fundus image. The more evident the neovascularization characteristics in the images, the better the network can learn to identify the lesions. Initially, the green channel is extracted from the RGB fundus images. This channel is selected because the blood vessels, including those associated with neovascularization, appear clearer in this channel than the red or blue channels [48], as shown in Figure 3. The blood vessel’s visibility is then improved by using Contrast Limited Adaptive Histogram Equalization (CLAHE) [49]. CLAHE adjusts the image contrast so that the foreground (blood vessels) became clearer than the background.

**Figure 4:**
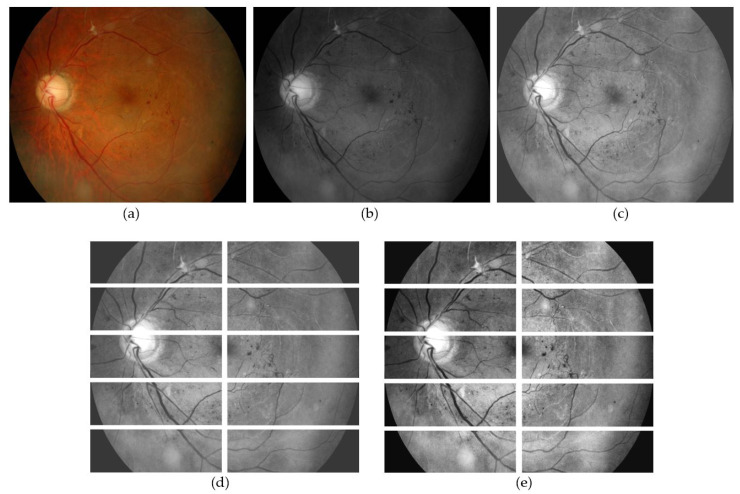
Image pre-processing steps. (**a**) input fundus image; (**b**) green channel extraction; (**c**) CLAHE is applied to the image; (**d**) the image is cropped into ten small patches; (**e**) image normalization is applied to the patches.

Each pre-processed fundus image is then divided into 10 smaller patches. The size of each patch is 400 × 1184 pixels. There is a total of 200 patches created from the 20 fundus images. Image normalization [50] is then applied to each patch to improve the visibility of the neovascularization vessels by normalizing the range of pixel intensity values within a patch. The resulting image patches are used for network training, validation, and testing. Fifty percent of the 200 image patches are chosen at random for training, 25 percent for validation, and the remaining 25 percent for testing. Figure 4 illustrates an example of a training image and output at each image pre-processing step.

**Figure 5:**
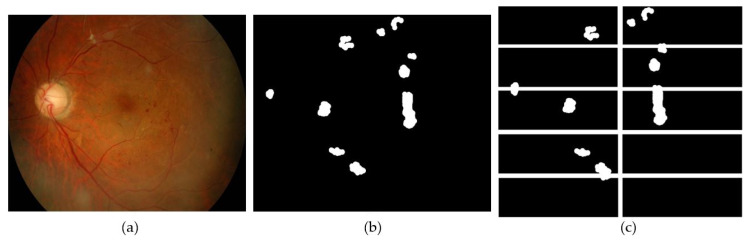
Ground truth cropping. (**a**) input image; (**b**) ground truth image; (**c**) ground truth was cropped into patches with each pixel corresponding to the image patches’ pixels in Figure 4e.

Each ground truth image is also subjected to the same cropping and divided into 10 smaller patches. During training, the network learns to identify each pixel as Neo or NotNeo based on its ground truth. The process of cropping ground truth is depicted in Figure 5.

Data augmentation is applied to the images in the training set to increase the number of training images. The augmentation process includes flipping the images horizontally and vertically. This increases the number of training images from 100 to 300. 

### 3.2. Network Design and Training

**Figure 6:**
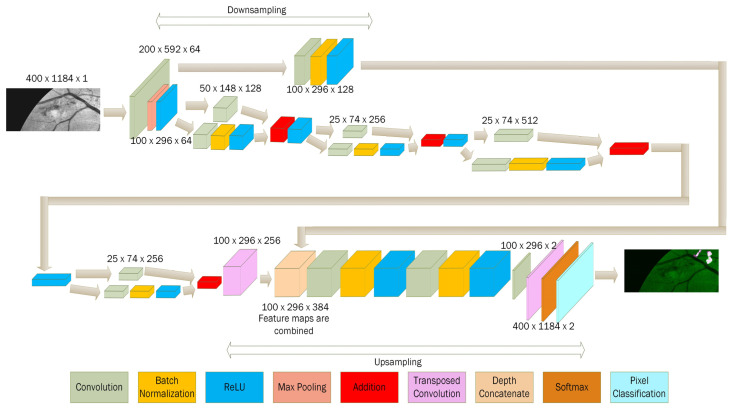
The structure of the network architecture.

A semantic segmentation convolutional neural network architecture is designed for learning the features of NotNeo and Neo pixels. This network is constructed using 42 layers. The layers include the convolution layer, max-pooling layer, batch normalization layer, and rectified linear unit layer. The structure of the network architecture is depicted in Figure 6.

A typical convolutional neural network used for neovascularization detection in other papers had only a single output [46]. A fully connected layer is used to classify images using the outputs of the convolution and pooling layers. However, this could only determine whether neovascularization is present in an image. It is unable to localize the lesion. To overcome this, semantic segmentation [51] is implemented in the proposed network. A pixel classification layer is used rather than a fully connected layer to achieve many outputs. The number of outputs is equal to the number of pixels in the image. Each pixel in the image is classified into one of two classes: Neo or NotNeo. As a result, neovascularization detection becomes more precise, with each pixel being scrutinized to detect and precisely locate the tiny vessels.

Due to the small size of the neovascularization vessels, smaller filters in the convolution layers may be preferred. However, the fundus images used in this study have a high resolution (2000 × 2368 pixels). Hence, instead of using the optimal 3 × 3 filter size, a 7 × 7 filter size is used. More pixels are considered when the feature map is constructed after the pixels pass through the first convolution layer. A 3 × 3 filter size is used for the subsequent convolution layers because the image has been downsampled, which reduces the image resolution. A 1 × 1 filter size is used when the image is downsampled to a low resolution, leaving few pixels available for convolution. Unlike U-Net [52], the first convolution layer used a 3 × 3 filter size due to the small size of the training images used in their test (512 × 512 pixels). 

The purpose of downsampling and upsampling is to reduce the amount of memory used while training. This expedites the training process and requires less memory while training. Following a convolution layer, batch normalization and the rectified linear unit layer are added. Batch normalization has the potential to accelerate the training process [53]. Therefore, placing it after the convolution layer can reduce training time. The batch normalization layer transforms each input in the current mini-batch by subtracting its mean and dividing it by its standard deviation. When the trained network makes predictions on a new image, the batch normalization layer uses the trained mean and variance to normalize the input. However, it requires many mini-batch sizes for training to effectively approximate the population mean and variance from the mini-batch. Our training images are 2000 × 2368 pixels in size, and the mini-batch size used is seven. As a result, the number of mini-batch sizes is sufficiently large enough to ensure that batch normalization runs efficiently. The rectified linear unit (ReLU) is used as the activation function [54]. ReLU is commonly used in a convolutional neural network and has been shown to provide better results than other nonlinear activation functions [55].

A depth concatenation layer that combined the feature maps produced by the first convolution layer with the feature maps produced by a transposed convolution layer is used in the first upsampling. This method will increase the number of feature maps available for learning after the first upsampling, allowing the network to learn more neovascularization features without additional training images. Thus, this approach can improve the neural network’s performance. As with U-Net, the first upsampling uses information from the previous downsampling to increase the resolution of feature maps used for learning. However, our proposed approach differs from the U-Net approach in that it employs depth concatenation to increase more feature maps. In contrast, the U-Net approach increases the resolution of the feature maps. The advantage of our approach, which utilizes the depth concatenation layer, is that we maintained the size of the feature maps rather than increasing their resolution, which conserves memory during training.

An “addition layer” is a layer that integrates inputs from multiple neural network layers element by element. This is accomplished by the pixel-by-pixel addition of two feature maps to create a new output feature map. This approach is advantageous because it preserves information from the input image to the network’s final few layers, ensuring that no information from the original input is lost during training [56]. The concept originated with ResNet [56], which is called a residual block. Addition layers are used in the proposed network architecture to preserve the information from the input image, allowing the original input image data to be carried throughout the network architecture.

**Figure 7:**
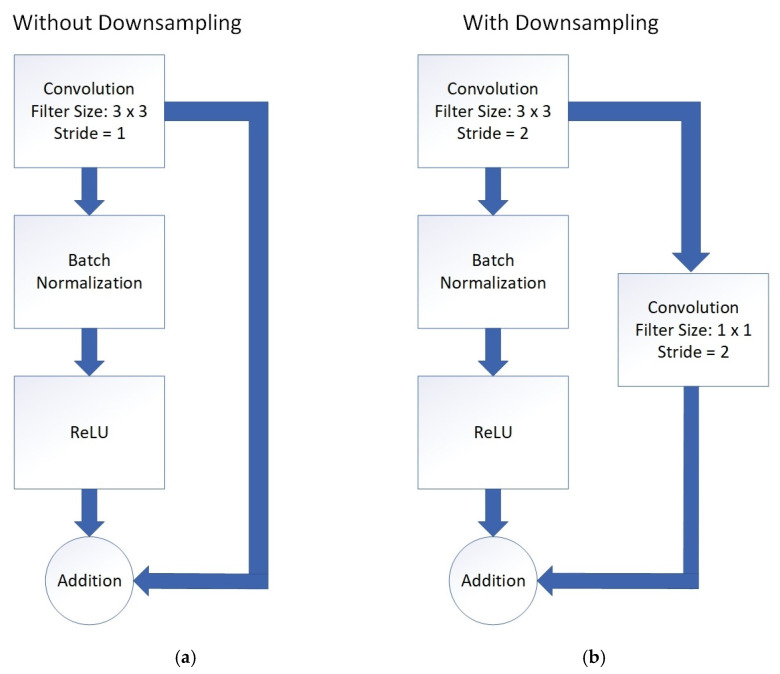
Application of addition layer to preserve information. (**a**) original residual block from ResNet; (**b**) modified residual block with downsampling.

Moreover, the residual block is modified so that the model simultaneously performed addition and downsampling. This is done by adding a 1 × 1 filter size convolution layer in the skip connection, as shown in Figure 7. Downsampling is accomplished by setting stride equal to 2 in the 3 × 3 and 1 × 1 convolution layers. The purpose of adding another convolution layer in the skipped connection is to perform downsampling in the skipped connection first before being added. This is because addition cannot be carried out if downsampling is only performed on the 3 × 3 convolution layer without performing another downsampling in the skipped connection due to the different resolutions of the two feature maps. The small filter size of 1 × 1 is used in the skipped connection’s convolution layer to prevent excessive filtering on the feature maps, ensuring that information is preserved while downsampling could occur concurrently.

The purpose of downsampling is to gradually reduce the image size in order to save on computational costs. Otherwise, training the network will consume a significant amount of memory. Therefore, downsampling is required to conserve memory during training. Upsampling is then used to restore the image to its original size, allowing each pixel in the original input image to be classified as neovascularization or non-neovascularization. Without downsampling, the resolution of feature maps will remain constant throughout the network architecture. Thus, the input size will be conserved until the end of the network layers. As a result of the increased parameter load, the network requires more memory to train. Therefore, downsampling is necessary to reduce the training parameters.

In the network training, the mini-batch size, epoch, momentum, and initial learning rate are set to 7, 10, 0.9, and 5 × 10^−4^, respectively. These values are obtained empirically from parameter tuning. The training is conducted using the training set and the validation set. Stochastic gradient descent with momentum as the optimizer was used to train the model. This optimizer determined the global minimum of the cross-entropy loss function with respect to weights as quickly as possible. The weight with the smallest loss represents the ideal weight for detecting neovascularization features in the dataset. During training, the weight was updated by measuring the loss after each mini-batch size. After reaching the global minima of the loss function, the training was terminated, and the optimal weight was determined. To prevent overfitting during the training, hold-out cross-validation was used to partition the dataset into a training set and a validation set.

The network will calculate the loss in the validation set after each mini-batch size during training. Once the loss on the validation set exceeds or equals the previously smallest loss, the network will automatically stop training. The number of times it can be greater than or equal to the previously smallest loss is referred to as validation patience. In the experiment, the validation patience was set to four. This value was obtained empirically. This prevents overfitting and allows the network to learn the optimal weight to identify neovascularization features rather than memorize each detailed feature in each image patch.

### 3.3. Image Segmentation and Performance Evaluation

After training is completed, the network is evaluated using the testing set. The network performs image segmentation by classifying each pixel in the test image as Neo or NotNeo. For performance evaluation, these classified pixels are compared to the ground truth images. To evaluate the network’s performance, accuracy, sensitivity, specificity, and precision are calculated.

Accuracy represents the correctly classified instances over the total number of instances. The equation of accuracy is shown below:

(1) $$Accuracy=\frac{TP+TN}{TP+TN+FP+FN}$$

True positive (*TP*) represents the pixels that are correctly classified as Neo. True negative (*TN*) refers to the pixels that are correctly classified as NotNeo. False positive (*FP*) represents the pixels that are incorrectly classified as Neo. False negative (*FN*) indicates the pixels that are incorrectly classified as NotNeo.

Aside from that, sensitivity is also useful in measuring an algorithm’s performance. Sensitivity represents the tendency of correctly classified instances. The equation of sensitivity is defined as below:

(2) $$Sensitivity=\frac{TP}{TP+FN}$$

Another vital performance metric is specificity. It measures the tendency of correctly classified negative instances. The equation of specificity is shown below:

(3) $$Specificity=\frac{TN}{TN+FP}$$

Precision is measured as the ratio of correctly detected positive samples to the total number of positive detection (either correctly or incorrectly detected). Precision is a metric that calculates how accurate the model is at classifying a sample as positive. The equation of precision is as shown below:

(4) $$Precision=\frac{TP}{TP+FP}$$

Dice similarity is a statistical measure to compute the similarity of two samples. The value ranges from 0 to 1, with 1 being the best result. It is commonly used to measure the performance of segmentation results. The equation of Dice similarity coefficient is given below:

(5) $$Dice=\frac{2\times TP}{2\times TP+FP+FN}$$

Jaccard similarity coefficient is another statistical measure to determine the similarity and diversity of sample sets. It is also used to evaluate the segmentation performance. The formula of the Jaccard similarity coefficient is defined as [57]:

(6) $$Jaccard=\frac{TP}{TP+FP+FN}$$

### 3.4. Performance Comparison

The performance of the proposed method is compared to other published works that also used convolutional neural networks for neovascularization detection to highlight the improvements made. However, the dataset used in this study is different from those used in the previous works. Therefore, to ensure a fair comparison, the methods described in other papers are implemented, and their performance in neovascularization detection is evaluated using the same dataset. 

Setiawan et al. [46] used pre-trained convolutional neural networks for neovascularization detection. Their proposed method is implemented and evaluated using the fundus images in this study. The tested networks are GoogLeNet [58], ResNet18 [56], ResNet50 [56], and AlexNet [59]. 

GoogLeNet, ResNet18, and ResNet50 require the same input size of 224 × 224 pixels in the first layer. However, the first layer of AlexNet needs an input size of 227 × 227 pixels. Hence, two sets of datasets are prepared with the required sizes using the twenty 2000 × 2368 pixels color fundus images. This is done by cropping the images into 1600 patches with a size of 224 × 224 pixels. In addition, 50% of the patches are allotted for the training set, 25% for the validation set, while the remaining 25% are used for the testing set. The 1600 patches were then resized to 227 × 227 pixels to form another dataset for AlexNet.

The training set and validation set images are fed into the pre-trained convolutional neural networks. Then, features are extracted from a fully connected layer (4096 from AlexNet, and 1000 from GoogLeNet, ResNet18, and ResNet50). The features are then used to train the SVM classifier. A total of four classifiers are trained, one for each pre-trained network’s features. Next, the testing set is subjected to the same procedure for feature extraction. Finally, the performances of the classifiers are evaluated using the features extracted from the testing set. 

The performance of the proposed method is also compared to a method by Hassan et al. [35], who used conventional image processing techniques for neovascularization detection. Their method is implemented and tested using the images used in this study. The obtained results are then compared to the results of the proposed method.

## 4. Results and Discussion

The proposed semantic segmentation network is implemented and trained in the Matlab R2019b platform. The proposed network is designed using the Deep Network Designer in Matlab’s Apps. Training of the network will require a long time to achieve good results. However, using the Stochastic Gradient Descent with Momentum (SGDM) optimizer, the global minima of the loss function, which represents the optimum weight for recognizing neovascularization pixels, can be discovered faster. The loss function used in the training process is the cross-entropy loss function. This function measured the total number of errors made in the training or validation set. The loss value indicates how well a model performed after each optimization iteration. The accuracy metric is used to calculate the algorithm’s output interpretably. After the model parameters are determined, the accuracy of the model is expressed as a percentage. It is a metric that indicates how close the model’s prediction is to the actual results. 

After the training is complete, the testing set is used to evaluate the performance of the network. The testing set contains images that the network has never seen before. The pixels from these images are fed into the proposed network. Each pixel is then categorized into one of the two categories: Neo or NotNeo. After the classification is complete, the number of true positives, true negatives, false positives, and false negatives are calculated by comparing each categorized pixel to its ground truth. These parameters are then used to determine the accuracy, sensitivity, specificity, precision, Jaccard coefficient, and Dice coefficient.

The proposed method segments the regions with neovascularization in the images, and the results from the above calculation measure the segmentation performance. However, other neovascularization detection methods to be compared in this study are based on image patch classification. The methods from Setiawan et al. [46] and Hassan et al. [35] can only detect whether neovascularization is present in an image patch. In order to have a fair comparison, the performance of the proposed method was also evaluated based on image patch classification. This is done by dividing the segmented output images from the testing set into patches of 200 × 296 pixels. Patches that contain neo pixels are considered positive images, while the rest are negative images. The same division is performed on the ground truth images. The performance metrics (accuracy, sensitivity, specificity, and precision) based on image patch classification are then calculated, and these values are used to compare with the results from Setiawan et al.’s [46] and Hassan et al.’s [35] methods.

**Figure 8:**
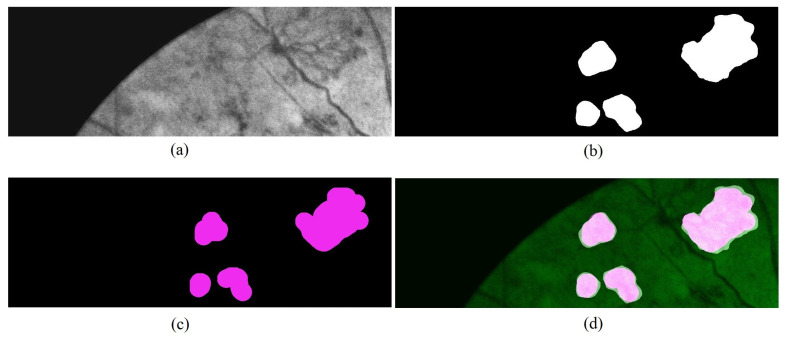
An example of an output image patch generated from the proposed network. (**a**) input image patch; (**b**) segmented Neo region by the network; (**c**) ground truth region; (**d**) the output image is generated by overlaying the segmented region and ground truth on the input image.

Figure 8 shows an example of an image patch and the output image generated by the proposed network. Figure 8a is the input image patch. The segmented neovascularization regions by the proposed network are shown in Figure 8b. These regions are compared to the ground truth image (Figure 8c). The final output image, as shown in Figure 8d, is obtained by overlaying the segmented regions and ground truth on the input image.

**Figure 9:**
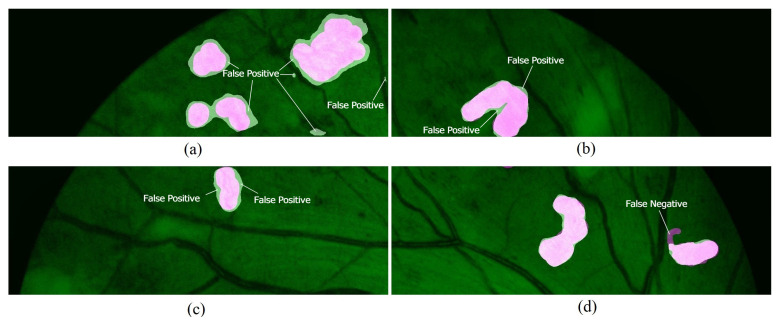
Examples of output images from the proposed network. Some false positives areas are labeled in (**a**–**c**). A false negatives area is labeled in (**d**).

**Figure 10:**
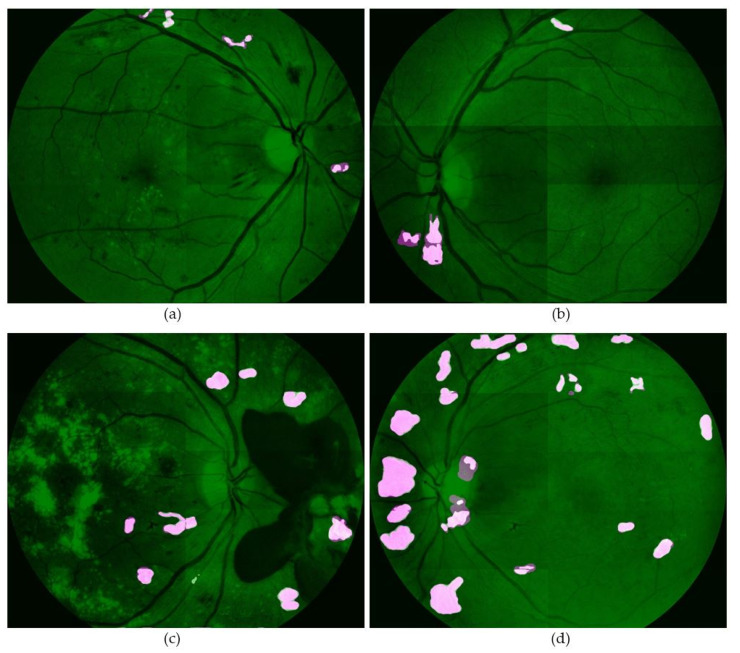
Several output images from the testing set are shown in (**a**–**d**). Note that the complete fundus images are obtained by combining the image patches.

Figure 9 shows four output images from the network. It can be observed that most of the segmented regions covered the ground truth in the images. This indicates that the proposed network is capable of detecting the vast majority of the Neo pixels. However, there are a few false positives near the edges of the ground truth, as labeled in Figure 9a–c). There are also some false negatives in several test images. They mostly occurred in images with small and narrow ground truth areas, as shown in Figure 9d. Figure 10 shows the results of several testing set image patches that have been combined to form the complete fundus images. 

Table 1 presents the evaluation results based on the performance metrics. The images used in the evaluation are from the testing set. The average results for image segmentation and image patch classification are given in the last two rows in the table.

For neovascularization segmentation, the obtained average accuracy is 0.9948. Sensitivity is equal to 0.8772 on average. This means that 87.72% of the Neo pixels are correctly identified as Neo. Specificity is 0.9976 on average. This indicates that 99.76% of the NotNeo pixels are correctly classified. The precision of 0.8696 demonstrates that 86.96% of the classified Neo pixels actually contain neovascularization. The segmentation results yielded an average Jaccard coefficient and Dice coefficient of 0.7643 and 0.8466, respectively. These results show that the proposed semantic segmentation network can achieve high accuracy, sensitivity, specificity, precision, and Dice coefficient. 

The average accuracy, sensitivity, specificity, and precision obtained for image patch classification are 0.9700, 0.9462, 0.9772, and 0.9263, respectively. This shows that, among the 400 image patches, 97% are correctly classified as Neo and NotNeo, 94.62% of the Neo patches are correctly classified, 97.72% of the NotNeo patches are correctly classified, and 92.63% of the classified Neo patches actually contain neovascularization. Certain test image patches were misclassified because the neovascularization features are not consistent across images. When the network learned the neovascularization features, it determined the optimal features that would produce the optimum result. Thus, any image patch containing neovascularization features that appear significantly different from the optimal learned features will be misclassified as non-neovascularization.

Another reason for the misclassification of certain image patches is that the neovascularization characteristics are overly complex. If the object is easy to identify, we can easily distinguish its features. However, due to the complexity of the tiny vessels in the retina, each neovascularization lesion appears quite differently in each image patch. As a result, it is challenging to avoid misclassification unless the neovascularization characteristics are consistent and straightforward, allowing for easy identification even with the naked eye.

To demonstrate the improvements made in neovascularization detection using the proposed method, its performance is compared with a recently published work by Setiawan et al. [46] that also used convolutional neural networks for neovascularization detection. To ensure fair performance comparison, the method described in the paper is implemented in this study (as explained in Section 3.4). Several pre-trained convolutional neural networks as proposed in the paper (GoogleNet, ResNet50, AlexNet, and ResNet18) are evaluated using our training and testing images. Another neovascularization detection method based on traditional image processing techniques by Hassan et al. [35] is also evaluated in this study to compare its performance with the proposed method. The results for each of the methods are presented in Table 2. These results are compared to the results of image patch classification from the proposed method.

The proposed network achieved the best results for accuracy, specificity, and precision among the evaluated methods. However, its sensitivity is slightly inferior (lower by 0.054 compared to the highest result). This demonstrates that the proposed model is effective at detecting neovascularization. 

In addition, the proposed deep learning model also has the advantage of segmenting the neovascularization pixels out of a fundus image, which is not possible with other methods. Other methods can only detect whether there is neovascularization in an image patch. It is unable to determine which pixels are associated with neovascularization. As a result, detecting neovascularization will be more precise using the proposed model by paying close attention to each pixel. Thus, using the proposed semantic segmentation convolutional neural network, neovascularization detection, and localization can both be accomplished automatically without additional effort.

## 5. Conclusions

This paper has presented a semantic segmentation convolutional neural network architecture for detecting neovascularization. Since neovascularization vessels are tiny, semantic segmentation is suggested. As a result of paying close attention to each pixel, neovascularization detection and localization via semantic segmentation will be more precise. Moreover, the proposed method is completely automated in detecting and localizing neovascularization lesions, which is not possible with a conventional convolutional neural network as proposed in other papers. The performance comparison results show that the proposed network outperformed other methods of neovascularization detection in terms of accuracy, specificity, and precision.

## Figures and Tables

**Table 1:** Performance of the proposed method (results from the testing set).

Image Number	Accuracy	Sensitivity	Specificity	Precision	Jaccard	Dice
1	0.9926	0.7330	0.9966	0.7626	0.5968	0.7475
2	0.9928	0.5841	0.9995	0.9536	0.5680	0.7245
3	1.0000	-	1.0000	-	-	-
4	1.0000	-	1.0000	-	-	-
5	1.0000	-	1.0000	-	-	-
6	0.9930	0.4845	0.9999	0.9837	0.4806	0.6492
7	1.0000	-	1.0000	-	-	-
8	1.0000	-	1.0000	-	-	-
9	1.0000	-	1.0000	-	-	-
10	1.0000	-	1.0000	-	-	-
11	1.0000	-	1.0000	-	-	-
12	0.9960	0.9811	0.9961	0.7188	0.7090	0.8297
13	1.0000	-	1.0000	-	-	-
14	1.0000	-	1.0000	-	-	-
15	1.0000	-	1.0000	-	-	-
16	1.0000	-	1.0000	-	-	-
17	0.9828	0.6749	0.9992	0.9781	0.6649	0.7987
18	1.0000	-	1.0000	-	-	-
19	0.9879	0.7187	0.9996	0.9859	0.7114	0.8313
20	1.0000	-	1.0000	-	-	-
21	0.9841	0.9960	0.9828	0.8623	0.8593	0.9243
22	0.9964	0.9795	0.9972	0.9415	0.9233	0.9601
23	0.9961	0.9822	0.9964	0.8132	0.8014	0.8897
24	0.9920	0.8949	0.9982	0.9700	0.8708	0.9309
25	0.9968	0.9799	0.9973	0.9071	0.8905	0.9421
26	1.0000	-	1.0000	-	-	-
27	1.0000	-	1.0000	-	-	-
28	1.0000	-	1.0000	-	-	-
29	1.0000	-	1.0000	-	-	-
30	0.9995	-	0.9995	0.0000	0.0000	0.0000
31	1.0000	-	1.0000	-	-	-
32	0.9958	0.9618	0.9972	0.9356	0.9021	0.9485
33	1.0000	-	1.0000	-	-	-
34	0.9969	0.9957	0.9969	0.8915	0.8881	0.9407
35	1.0000	-	1.0000	-	-	-
36	1.0000	-	1.0000	-	-	-
37	0.9891	0.8630	0.9946	0.8747	0.7681	0.8688
38	0.9906	0.8363	0.9988	0.9744	0.8184	0.9001
39	0.9983	0.9501	0.9992	0.9606	0.9145	0.9553
40	0.9923	0.9972	0.9922	0.8004	0.7986	0.8880
41	0.9855	0.9869	0.9854	0.8585	0.8488	0.9182
42	0.9840	0.9360	0.9874	0.8422	0.7963	0.8866
43	0.9945	0.9844	0.9952	0.9311	0.9175	0.9570
44	0.9959	0.9409	0.9973	0.8925	0.8452	0.9161
45	0.9439	0.7209	0.9906	0.9417	0.6901	0.8166
46	1.0000	-	1.0000	-	-	-
47	0.9760	0.8655	0.9898	0.9137	0.8001	0.8889
48	0.9958	0.9187	0.9987	0.9643	0.8885	0.9410
49	0.9927	0.9633	0.9955	0.9523	0.9190	0.9578
50	1.0000	-	1.0000	-	-	-
Average (for image segmentation results)	0.9948	0.8772	0.9976	0.8696	0.7643	0.8466
Average (for image patch classification results)	0.9700	0.9462	0.9772	0.9263	-	-

**Table 2:** Performance comparison of the proposed method with other neovascularization detection methods. The best values are indicated in bold.

Method	Accuracy	Sensitivity	Specificity	Precision
Setiawan et al. [46] (GoogLeNet with SVM)	0.6650	0.9850	0.3450	0.6006
Setiawan et al. [46] (ResNet50 with SVM)	0.9200	0.9950	0.8450	0.8652
Setiawan et al. [46] (AlexNet with SVM)	0.8325	**1.0000**	0.6650	0.7491
Setiawan et al. [46] (ResNet18 with SVM)	0.7525	**1.0000**	0.5050	0.6689
Hassan et al. [35]	0.6502	0.7150	0.5766	0.6573
Proposed method	**0.9700**	0.9462	**0.9772**	**0.9263**

## Data Availability

Not applicable.
